# Innovative Application of Medicinal Insects: Employing UHPLC-MS, Bioinformatics, In Silico Studies and *In Vitro* Experiments to Elucidate the Multi-Target Hemostatic Mechanism of *Glenea cantor* (Coleoptera: Cerambycidae) Charcoal-Based Medicine

**DOI:** 10.3390/ph18040479

**Published:** 2025-03-27

**Authors:** Bangyu Zhong, Wen Zhang, Liangshan Ming, Qimeng Fan, Lei Zhang, Hongyu Lai, Genwang Huang, Hongning Liu, Zishu Dong

**Affiliations:** 1 National Key Laboratory for the Modernization of Classical and Famous Prescriptions of Chinese Medicine, Jiangxi University of Chinese Medicine, Nanchang 330004, China; zhongbangyu@jxutcm.edu.cn (B.Z.); zhangwen45@jxutcm.edu.cn (W.Z.); jazmaster@163.com (L.M.); 20201018@jxutcm.edu.cn (Q.F.); zhanglei54@jxutcm.edu.cn (L.Z.); laihongyu@jxutcm.edu.cn (H.L.); huanggenwang@jxutcm.edu.cn (G.H.); 2 Research Center for Differention and Development of TCM Basic Theory, Jiangxi University of Chinese Medicine, Nanchang 330004, China; 3 College of Pharmacy, Jiangxi University of Chinese Medicine, Nanchang 330004, China; 4 Advanced Research Institute, Jiangxi University of Chinese Medicine, Nanchang 330004, China

**Keywords:** UHPLC-MS, hemostasis, network pharmacology, molecular docking, molecular dynamics simulation, natural products, biopharmaceuticals

## Abstract

**Background:** Longhorn beetles, a widely recognized group of Chinese traditional medicinal insects, are characterized by their notable hemostatic properties. However, the comprehensive understanding of their medicinal potential has been hindered by the limitations of current research methodologies. **Methods:** This study focuses on the species *Glenea cantor* (Fabricius), which can produce several generations per year, and introduces a novel method using microwave carbonization techniques. By employing an in vitro coagulation test, UHPLC-MS, network pharmacology, molecular docking, and molecular dynamics simulation, the hemostatic efficacy and mechanism of action of Glenea cantor charcoal medicine (GC-CM) were thoroughly studied. **Results:** In vitro coagulation tests showed that GC-CM significantly reduced the activated partial thromboplastin time (APTT) and prothrombin time (PT), indicating its ability to enhance the coagulation cascade and preliminarily confirming its hemostatic efficacy (*p* < 0.01 vs. blank control group). The analysis revealed that GC-CM comprises 453 components, including 137 bioactive components with high human utilization. After predictions via databases such as SwissTargetPrediction and deduplication, 215 targets linked to hemostatic specificity were identified. These targets regulate signaling pathways such as platelet activation, complement and coagulation cascades, and cGMP-PKG. Molecular docking demonstrated strong affinities between key targets such as SRC and PIK3R1 and compounds such as 2′,6′-dihydroxy 4′-methoxydihydrochalcone, and 1-monolinoleoyl-rac-glycerol (binding energy < −5 kcal/mol). Molecular dynamics simulations show good binding capacity between core components and targets **Conclusions:** The aim of this study was to elucidate the material basis and mechanism of the hemostatic efficacy of GC-CM, offering a model for exploring other insect-based medicinal resources.

## 1. Introduction

In recent years, substantial advancements have been achieved in the research of both natural and synthetic hemostatic agents; however, these agents continue to encounter numerous limitations. Natural hemostatic agents, such as fibrin glue and chitosan, exhibit biocompatibility; nevertheless, their utilization is frequently constrained by high costs and ethical concerns, as well as supply chain challenges associated with human-derived fibrin glue [1,2]. Meanwhile, synthetic hemostatic agents, including oxidized cellulose and polyethylene glycol hydrogel, are chemically modified to improve hemostatic efficacy. However, they may induce tissue rejection or increase the risk of thrombosis, and their mechanisms of action are predominantly limited to a single target. This limitation poses challenges in addressing the clinical needs of patients with complex coagulation disorders [3,4]. In light of these issues, hemostatic agents derived from medicinal insects are increasingly demonstrating their potential advantages.

Medicinal insects, distinguished by their rich diversity of natural compounds and unique pharmacological activities, play a critical role in the therapeutic management of various diseases [5,6]. The family Cerambycidae, commonly known as longhorn beetles, belongs to the order Coleoptera and the suborder Polyphaga and is one of the recorded traditional Chinese medicines. This globally distributed family encompasses a substantial diversity of species, with over 36,000 species documented, establishing its significance within the class Insecta [7]. In the literature of Eastern traditional medicine, longhorn beetles are recognized for their distinctive therapeutic properties and efficacy [8]. Contemporary scientific research has further substantiated the considerable medicinal potential of longhorn beetles in the realms of anti-inflammatory, analgesic, and hemostatic applications [9]. Research has demonstrated that high-temperature carbonization can enhance the hemostatic efficacy of traditional Chinese medicine by facilitating the preservation or augmentation of active compounds [10]. Given the hemostatic properties of longhorn beetles, this technique may be a viable approach for effectively harnessing and enhancing their medicinal value.

Following a comparable high-temperature carbonization process, traditional Chinese medicine is transformed into a distinct category of therapeutic agent known as carbonized Chinese medicine, or carbon medicine, which exhibits a broad spectrum of pharmacological effects. Presently, more than 70 varieties of carbon medicine are in use. The established clinical efficacy of these medicines serves as the primary impetus for their ongoing preservation and utilization, with their most prevalent application being in the domain of hemostatic treatment [11]. The hemostatic response in humans is a complex physiological process that involves cellular and molecular components within the blood, encompassing platelets, coagulation factors, and regulatory proteins [12]. Building on the unique efficacy of charcoal-based medicine and the development of alternative insect-derived pharmaceuticals alongside empirical applications in the medical field [13,14], this study established a microwave carbonization process specifically for longhorn beetles, with its mechanism of action elucidated through modern and well-established systematic biological methodologies.

Network pharmacology, an established systems biology approach, systematically maps interactions between drugs and biological targets to predict multi-target mechanisms [15]. By integrating bioinformatics resources and computational models [16], this methodology transcends reductionist target analysis, instead constructing holistic drug-action networks that decode molecular-level therapeutic effects. Its applications span drug discovery (screening novel candidates, optimizing structures, predicting side effects) [17] and mechanistic deconvolution across genetic, proteomic, and metabolic hierarchies [18]. Researchers often combine network pharmacology with molecular docking (assessing drug–target affinity [19,20,21]) and molecular dynamics simulations (validating binding stability [22]) to strengthen predictive reliability. This triad of computational techniques [23,24]—network-based target mapping, docking, and dynamics—forms a robust framework for evaluating drug efficacy/safety early in development, accelerating multi-target drug design and personalized medicine.

**Figure 1:**
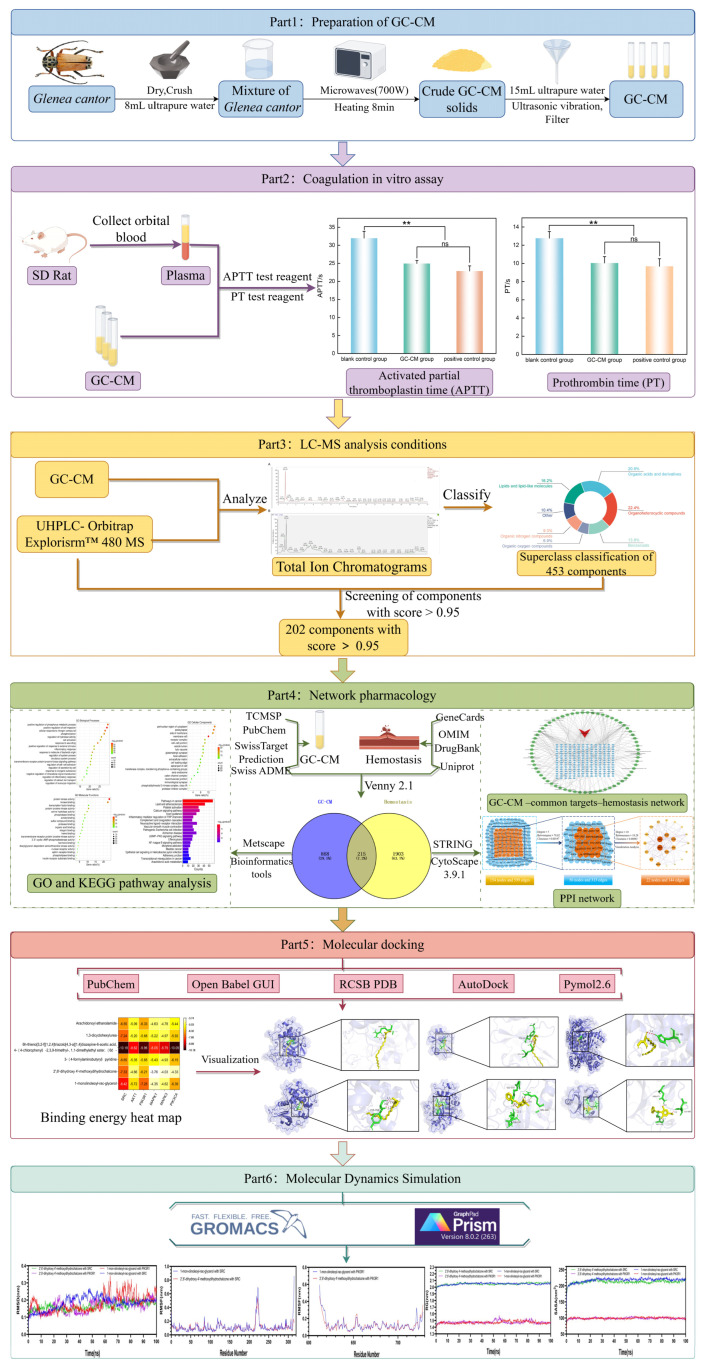
Workflow diagram of the hemostatic effect and mechanism of GC-CM.

The research subject of this study is the beetle *Glenea cantor*, a destructive forest pest, which breeds multiple generations per year [25]. The results of current research findings on the medicinal properties of longhorn beetles corroborate ancient literature, indicating significant potential for its application in medicine. Nevertheless, current methodologies for investigating the medicinal attributes of longhorn beetles remain constrained, predominantly focusing on the molecular level without thoroughly examining the underlying mechanisms of action. This limitation significantly hampers a comprehensive exploration of the longhorn beetles’ medicinal potential. A mature research method for the basic pharmacodynamics of traditional Chinese medicine is needed to systematically predict the potential mechanism of action of longhorn beetle charcoal medicine, thereby increasing drug development’s efficiency and success rate. This study presents the development of an innovative charcoal-based medicinal compound derived from longhorn beetles, utilizing an optimized microwave-assisted carbonization protocol. The research employed in vitro coagulation assays, ultra-high-performance liquid chromatography–mass spectrometry (UHPLC-MS), network pharmacology, molecular docking, and molecular dynamics simulations to elucidate the material basis of the hemostatic effect of GC-CM. Furthermore, the study aimed to preliminarily uncover the pharmacodynamic effects and mechanisms of action, thereby advancing the application of established Chinese medicine efficacy analysis techniques in investigating the therapeutic potential of medicinal insects. It offers novel perspectives that contribute to advancing research and development in exploring additional medicinal insect resources. The workflow diagram is shown in Figure 1.

## 2. Results

### 2.1. In Vitro Coagulation Assay Results of GC-CM

**Figure 2:**
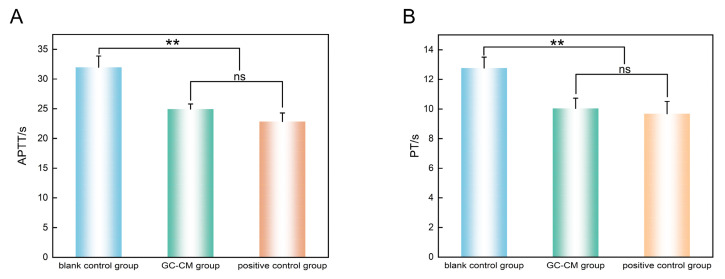
Results of the effect of GC-CM on APTT and PT. (**A**) Activated partial thromboplastin time (APTT). (**B**) Prothrombin time (PT). ** *p* < 0.01 vs. blank control group. ns, not significant. Data are expressed as means ± SD (n = 3).

The results are presented as means ± standard deviation (SD) in Table 1, and the corresponding bar chart analysis is illustrated in Figure 2. The reduction in activated partial thromboplastin time (APTT) by 21.9% (24.93 ± 0.85 s compared to 31.97 ± 1.90 s in the control group) and the 21.4% shortening of prothrombin time (PT) (10.04 ± 0.70 s versus 12.77 ± 0.73 s) closely approximated the efficacy observed with the positive control, which recorded values of 22.86 ± 1.42 s for APTT and 9.68 ± 0.83 s for PT. These findings indicate that GC-CM significantly reduces activated partial thromboplastin time (APTT) and prothrombin time (PT) (*p* < 0.01).

### 2.2. UHPLC-MS Analysis

**Figure 3:**
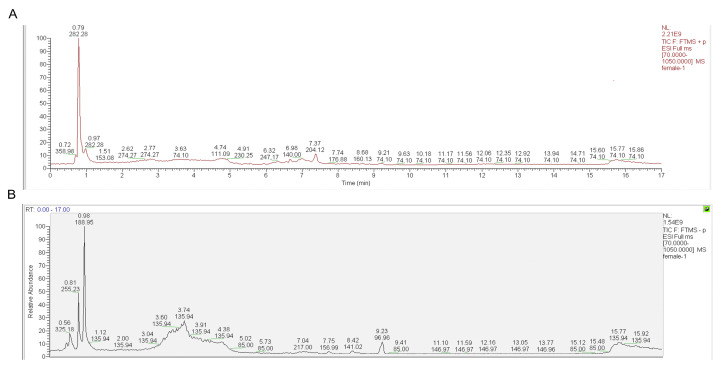
Total ion chromatogram of GC-CM. (**A**) Positive ion chromatogram of GC-CM. (**B**) Negative ion chromatogram of GC-CM.

**Figure 4:**
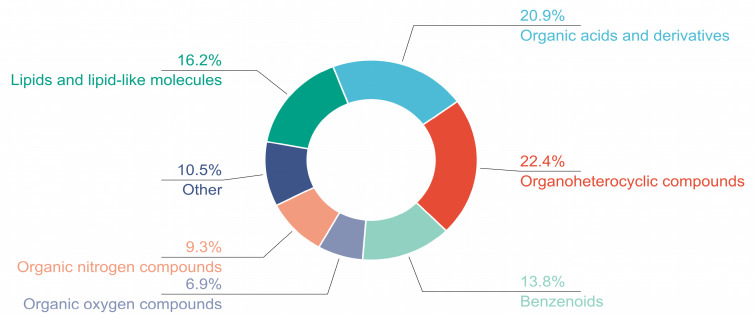
Superclass classification of 453 components.

Under the conditions described in Section 4.6, full-scan analysis of the GC-CM component samples was performed in both positive and negative ion modes, yielding total ion chromatograms (Figure 3A,B). A total of 453 components were detected, with their superclass classifications shown in Figure 4, primarily including organoheterocyclic compounds, organic acids and derivatives, and lipids and lipid-like molecules. From this dataset, 202 components with a score > 0.95 were selected for further analysis, as detailed in Supplementary Table S1.

### 2.3. Active Components of GC-CM and Its Potential Targets Related to Hemostasis

Through comprehensive searches on the TCMSP database and the Swiss ADME platform, we selected 137 components that can be effectively absorbed by the human body and possess potential activity. Using the SwissTargetPrediction database for predictions and after deduplication, 1083 GC-CM action targets were obtained. In the prediction results for hemostatic targets, 1985, 200, and 8 targets were obtained from the GeneCards, OMIM, and Drugbank databases, respectively. Duplicates were removed after combining the results from these three databases, identifying 2118 potential hemostatic-related targets.

### 2.4. The “Active Ingredient–Intersecting Target–Hemostasis” Network and Key Components of GC-CM

**Figure 5:**
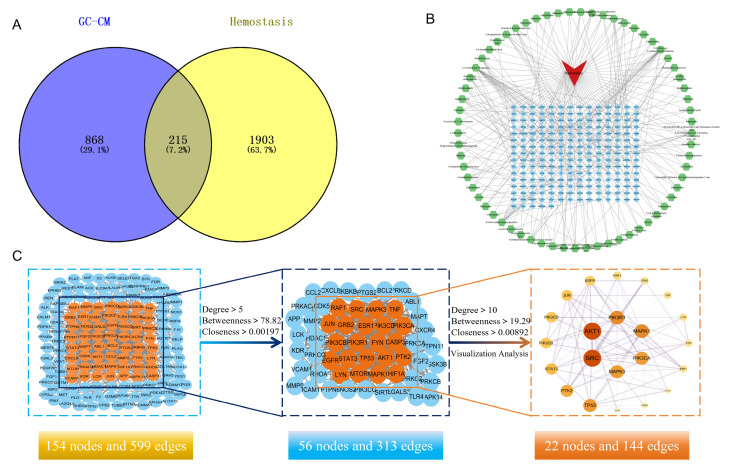
Network pharmacology analysis of GC-CM for hemostasis. (**A**) Venn diagram of GC-CM and hemostatic intersection targets. (**B**) GC-CM active ingredient–common target–hemostasis network. (**C**) PPI network of GC-CM’s targets for promoting hemostasis.

Using Venny 2.1, we identified 215 intersection targets between GC-CM and hemostasis (Figure 5A). The “active ingredients of GC-CM–intersecting target–hemostasis” network (Figure 5B) was constructed using CytoScape 3.9.1 to elucidate the potential molecular mechanisms between the 137 active components and the 215 intersecting targets. The connecting lines between nodes represent the links between interacting active components, intersecting targets, and hemostasis. The higher the density of the lines, the greater the degree of association between nodes. Based on the Cytoscape network topology analysis, using the degree value as the reference value, it was concluded that the main active components of GC-CM affecting hemostasis included 1-monolinoleoyl-rac-glycerol, 2′,6′-dihydroxy 4′-methoxydihydrochalcone, 3-(4-formylaminobutyryl)pyridine, etc. Table 2 shows the top 20 components in terms of degree value.

### 2.5. PPI Network

The analysis conducted using Centiscape 2.2 delineated two screening criteria—for the initial round, Degree Centrality (DC) > 5, Betweenness Centrality (BC) > 78.82, and Closeness Centrality (CC) > 0.00197, and for the subsequent round, DC > 10, BC > 19.29, and CC > 0.00892—resulting in the identification of 22 core targets (Figure 5C); their topological parameters are shown in Table 3. Within this network, nodes positioned closer to the center exhibit a darker color and larger diameter, signifying a higher network degree value for these targets. The connections between nodes elucidate the interaction relationships among the targets, with thicker lines and deeper colors signifying higher combined scores and enhanced interaction capabilities between the targets. The protein–protein interaction (PPI) analysis identifies SRC, AKT1, PIK3R1, MAPK1, MAPK3, and PIK3CA as significant targets.

### 2.6. GO and KEGG Pathway Analysis of GC-CM with Hemostatic Intersection Targets

**Figure 6:**
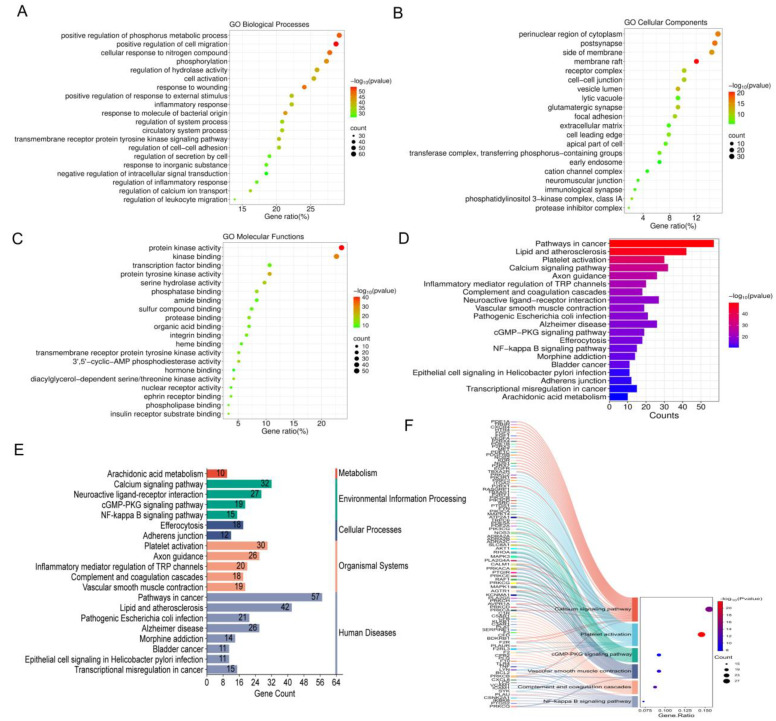
The GO and KEGG enrichment analysis of intersection targets between GC-MC and hemostasis. (**A**) Biological process (BP) enrichment analysis of intersection targets between GC-MC and hemostasis. (**B**) Cellular component (CC) enrichment analysis of intersection targets between GC-MC and hemostasis. (**C**) Molecular function (MF) enrichment analysis of intersection targets between GC-MC and hemostasis. (**D**) KEGG enrichment analysis of intersecting targets between GC-MC and hemostasis. (**E**) Classification of KEGG enrichment analysis. (**F**) Sankey vs. dot plot of genes enriched in KEGG pathway analysis results.

The information exported from the Metscape platform was analyzed, and the top 20 results with the smallest *p*-value were visualized. This analysis covered various aspects of Gene Ontology (GO), including biological processes (BP), cellular components (CC), and molecular functions (MF). The BP results indicate that GC-CM is primarily involved in human metabolism’s biological processes: positive regulation of phosphorus metabolic process, positive regulation of cell migration, cellular response to nitrogen compounds, response to wounding, and phosphorylation (Figure 6A). The CC results emphasize the association between GC-CM and components, including the perinuclear region of cytoplasm, postsynapse, side of membrane, and membrane raft (Figure 6B). The MF analysis shows that GC-CM mainly regulates hemostasis by influencing functions including protein kinase activity, kinase binding, and protein tyrosine kinase activity (Figure 6C). The KEGG enrichment analysis revealed that highly enriched pathways include platelet activation, calcium signaling pathway, complement and coagulation cascades, vascular smooth muscle contraction, cGMP-PKG signaling pathway, and NF-kappa B signaling pathway (Figure 6D). Figure 6F illustrates the genes enriched within these six pivotal signaling pathways. The core targets AKT1, MAPK1, and MAPK3 are prominently enriched across multiple pathways. Furthermore, the six core targets exhibiting the highest degree values within the protein–protein interaction (PPI) network are all associated with platelet activation. Figure 6E categorizes the 20 enriched pathway results presented in Figure 6D, revealing that the majority pertain to Human Diseases, Organismal Systems, and Environmental Information Processing.

### 2.7. Verification with Molecular Docking

**Figure 7:**
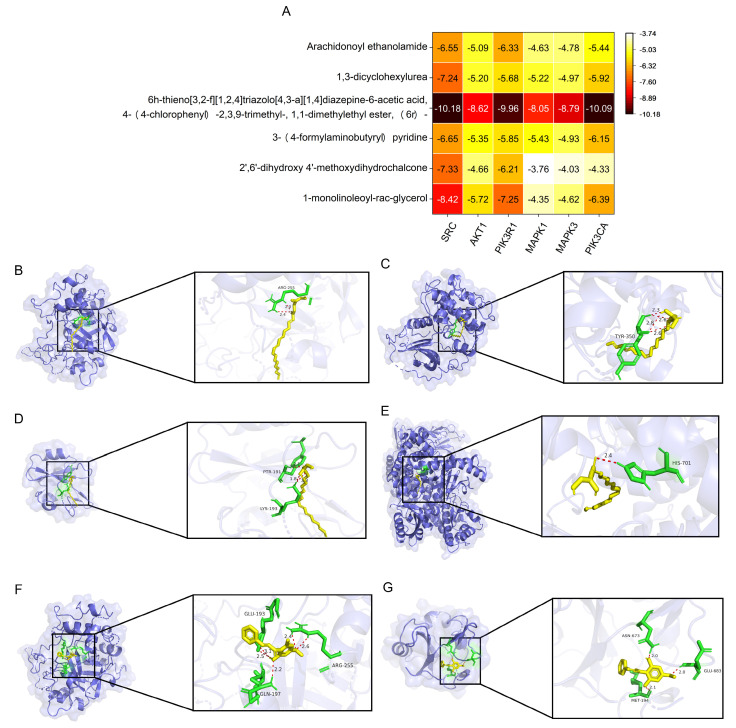
Visual analysis of molecular docking. (**A**) Heat map of key targets and the core active ingredients of GC-CM molecular docking scoring. (**B**) 1-mon-olinoleoyl-rac-glycerol–SRC. (**C**) 1-monolinoleoyl-rac-glycerol–AKT1. (**D**) 1-mon-olinoleoyl-rac-glycerol–PIK3R1. (**E**) 1-mon-olinoleoyl-rac-glycerol–PIK3CA. (**F**) 2′,6′-dihydroxy 4′-methoxydihydrochalcone–SRC. (**G**) 2′,6′-dihydroxy 4′-methoxydihydrochalcone–PIK3R1.

The top six compounds with the highest degree were docked with the top six proteins, and their corresponding binding energies were calculated. The selectivity and function of the ligands were evaluated through docking simulations to assess their binding potential based on the comprehensive characteristics of proteins and ligands, aiming to ensure prediction accuracy and reliability. Binding energy is a key indicator of docking results; typically, a lower binding energy value indicates a more stable protein–ligand interaction: a binding energy < −4.25 kcal/mol suggests moderate binding activity, <−5.0 kcal/mol indicates good binding activity, and <−7.0 kcal/mol suggests strong binding activity [26]. Among the 36 docking results, 10 pairs (27.8%) exhibited binding energies less than −7.0 kcal/mol, 26 pairs (72.2%) demonstrated binding energies below −5.0 kcal/mol, and 34 pairs (94.4%) had binding energies under −4.25 kcal/mol (Figure 7A), indicating that most targets exhibit good binding activity with the components. Notably, the compound 6h-thieno [3,2-f][1,2,4]triazolo [4,3-a][1,4]diazepine-6-acetic acid, 4-(4-chlorophenyl)-2,3,9-trimethyl-, 1,1-dimethylethyl ester, (6r)- exhibited binding energies less than −7.0 kcal/mol with all six proteins; the above results confirm that GC-CM works well in hemostasis. To further investigate the interactions between core ligands and primary gene targets, we selected six docking results pertinent to the two principal core components—1-monolinoleoyl-rac-glycerol and 2′,6′-dihydroxy 4′-methoxydihydrochalcone—based on their binding energy and network degree values, for three-dimensional spatial environment imaging. 1-monolinoleoyl-rac-glycerol establishes two, four, one, two and one hydrogen bond with the amino acid residues ARG-255, TYR-350, PTR-191, LYS-193, and HIS-701 of SRC, AKT1, PIK3R1, and PIK3CA, respectively, indicating stable binding interactions (Figure 7B–E). Similarly, 2′,6′-dihydroxy 4′-methoxydihydrochalcone forms five hydrogen bonds with three amino acid residues (ARG-255, GLU-193, and GLN-197) of SRC and three hydrogen bonds with three amino acid residues (ASN-673, GLU-683, and MET-194) of PIK3R1, suggesting a stable binding affinity (Figure 7F,G). These results further demonstrate that the main active components of GC-CM have good affinity for the core targets, reflecting that GC-CM can regulate the process of hemostasis through these targets.

### 2.8. Verification with Molecular Dynamics Simulation

**Figure 8:**
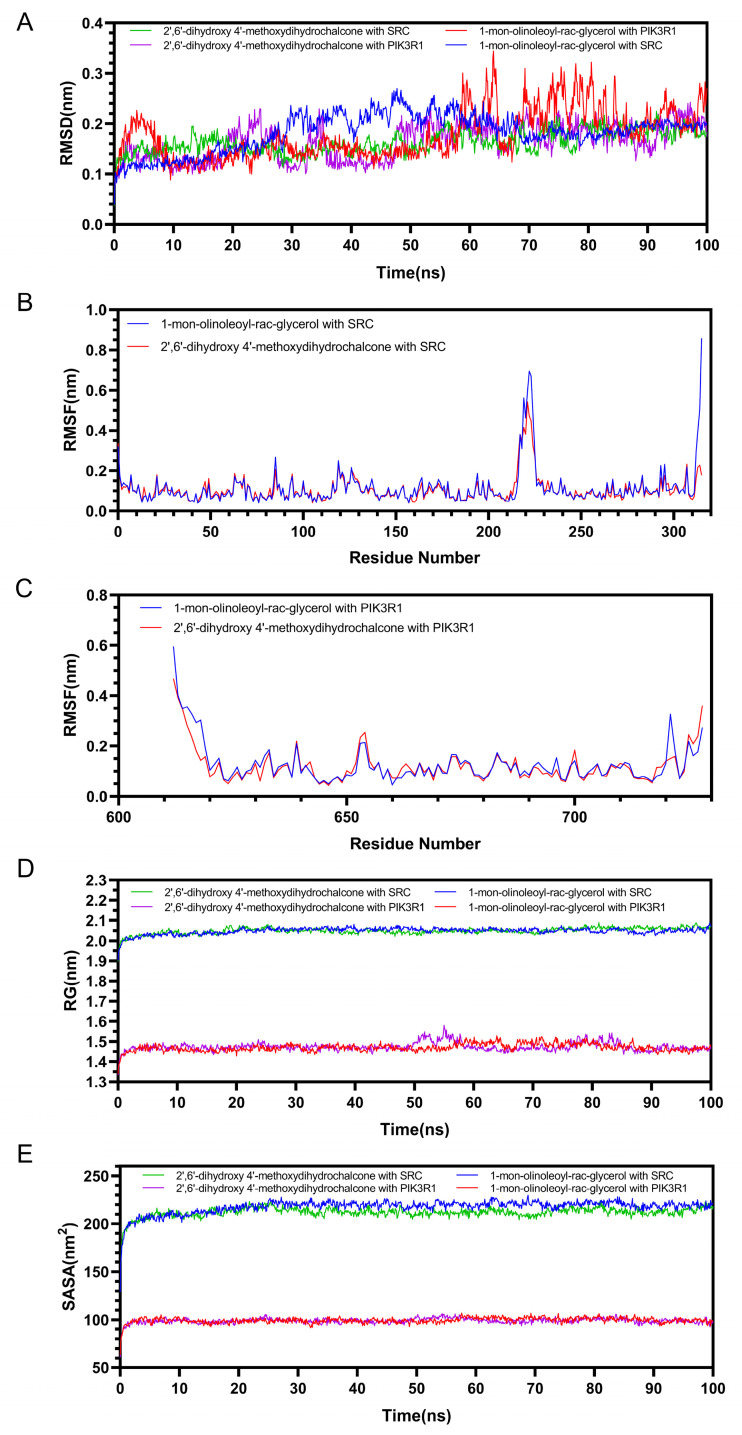
Molecular dynamics simulations of SRC and PIK3R1 with the core components 1-mono-olinoleoyl-rac-glycerol and 2′,6′-dihydroxy 4′-methoxydihydrochalcone. (**A**) RMSD. (**B**) The RMSF of SRC. (**C**) The RMSF of PIK3R1. (**D**) RG. (**E**) SASA.

RMSD (root mean square deviation) values indicate the extent of conformational fluctuations in proteins and are instrumental in evaluating the stability of ligand binding to target proteins. In the four complexes depicted in Figure 8A, the RMSD values of the protein backbone exhibited minor fluctuations within the range of 0.2 nm to 0.3 nm, suggesting that the complexes maintain a relatively stable state throughout the simulation. RMSF (root mean square fluctuation) provides insights into the variability of protein amino acid residues. Figure 8B,C illustrate the fluctuations of amino acid residues in the proteins SRC and PIK3R1 during the simulation. Despite certain regions exhibiting fluctuations, the overall structure of the complexes remains relatively stable, indicating that most of the protein regions preserve substantial rigidity following ligand binding. The radius of gyration (Rg) is commonly used to assess the overall compactness of a protein and can also indicate molecular affinity. The solvent-accessible surface area (SASA) characterizes the interaction between the protein molecule and the surrounding water molecules, thereby reflecting the degree of ligand exposure to the solvent and indirectly indicating the extent of protein encapsulation of the ligand. The plots of Rg and SASA revealed no significant conformational alterations, suggesting stable ligand–protein binding (Figure 8D,E).

## 3. Discussion

Using insects as medicinal resources represents an underexplored frontier in the sustainable bioeconomy, particularly for phytophagous pests, whose ecological costs could be offset through value-added applications [27]. Notably, members of the Cerambycidae family, including *Glenea cantor*, have demonstrated pharmacological potential; historical records indicate that certain longhorn beetles were traditionally used to treat trauma, with their bioactive components showing modulatory effects on coagulation pathways [9]. Recent advances in charcoal medicine preparation technology have further unlocked the hemostatic potential of insect-derived materials by enhancing hemostatic constituents through pyrolysis-induced structural modifications [11]. In this context, our study pioneers the transformation of *Glenea cantor*—a destructive borer of kapok trees—into a novel hemostatic agent. This approach not only aligns with the principles of the circular economy by repurposing pest species but also holds potential applications in emergency medicine.

APTT and PT are the most frequently utilized parameters for assessing coagulation status. In vitro coagulation assays have demonstrated that GC-CM significantly reduces APTT and PT, suggesting that GC-CM modulates the levels and activities of coagulation factors XII, V, VII, and X via both intrinsic and extrinsic coagulation pathways, thereby enhancing coagulation [28]. Furthermore, these findings corroborate the involvement of GC-CM in regulating pathways such as the complement and coagulation cascades, as identified through enrichment analysis. Research conducted by Li et al. [28,29] investigating the hemostatic properties of peony bark charcoal demonstrated that certain active constituents within peony bark charcoal are capable of activating coagulation factors XII and X, subsequently leading to a reduction in APTT and PT in rat models. These findings align with the results observed in the present study.

Hemostasis progresses through two distinct phases: primary hemostasis, which involves vascular contraction and platelet adhesion/activation, and secondary hemostasis, characterized by the convergence of intrinsic and extrinsic coagulation cascades leading to fibrin clot formation [30,31,32,33]. Activated platelets play a crucial role in enhancing thrombin generation by providing an anionic phospholipid surface for the assembly of coagulation factors [34,35,36]. KEGG enrichment analysis revealed that the targets of GC-CM are primarily associated with platelet activation, coagulation cascades, and vascular smooth muscle contraction. This pattern is consistent with the mechanisms of action of charcoal medicine as documented by Wang et al. [37] and Li et al. [29], thereby confirming GC-CM’s role in enhancing hemostasis across both phases.

Platelet activation and adhesion during hemostasis depend on tyrosine kinase cascades, particularly SRC—a proto-oncogene critical for platelet signaling and proplatelet formation [38]. In resting platelets, SRC remains inactive through binding to integrin αIIbβ3′s β-subunit. Fibrinogen-induced αIIbβ3 activation triggers SRC phosphorylation, initiating outside-in signaling [39]. Concurrently, the GPIb-IX-vWF adhesion complex recruits AKT1/AKT2 to mediate early platelet activation [40]. Our protein–protein interaction (PPI) network identified SRC and AKT1 as top-ranked core targets, suggesting that GC-CM’s hemostatic efficacy relies on modulating these kinases. Supporting this, Gene Ontology (GO) enrichment revealed that GC-CM-associated targets are significantly linked to tyrosine kinase activity and binding, corroborating its regulatory role in platelet signaling cascades.

The protein–protein interaction (PPI) network analysis identified MAPK1 and MAPK3, members of the ERK subfamily integral to MAPK signaling, as pivotal regulatory nodes [41]. Notably, the p38 MAPK pathway directly mediates platelet activation via calcium-dependent phosphorylation of phospholipase A2 (PLA2) at Ser505, triggering thromboxane A2 (TxA2) synthesis to amplify platelet responses [42,43]. Integrative GO/KEGG analyses suggest that GC-CM modulates calcium flux and MAPK1/MAPK3 activity, thereby potentiating p38 MAPK-driven hemostasis—a mechanism corroborated by established calcium signaling roles in coagulation [44,45]. Concurrently, GC-CM targets the components of the PI3K complex, specifically PIK3R1 (regulatory subunit) and PIK3CA (catalytic subunit), which are pivotal in orchestrating the PI3K-AKT signaling pathway essential for platelet adhesion and aggregation [46,47]. The phosphorylation of SRC initiates this signaling cascade, with AKT1 functioning as the terminal effector [48,49,50,51,52]. Although our protein–protein interaction (PPI) network analysis confirms that SRC, AKT1, PIK3R1, and PIK3CA are central to the hemostatic action of GC-CM, the intricate dynamics of their interactions warrant further investigation. Strikingly, the 22 core targets identified in this network exhibited a high degree of similarity to those reported by Zheng et al. [53] in their investigation of the hemostatic mechanism of gardenia, thereby further validating the accuracy of the present study.

KEGG enrichment analysis has identified the cGMP-PKG and NF-κB pathways as central to the hemostatic action of GC-CM. The cGMP-PKG axis plays a crucial role in regulating platelet activation and inhibition by mediating nitric oxide-induced suppression of P-selectin expression and platelet rigidity [54,55], thereby maintaining coagulation homeostasis. In contrast, although the NF-κB pathway is traditionally associated with inflammation, it indirectly enhances coagulation through cytokine-driven thrombin generation during inflammatory responses [56]. While these pathways collectively elucidate GC-CM’s dual modulation of platelet activity and the interplay between inflammation and coagulation, the precise molecular interactions involved require further mechanistic insights.

This study utilized the “GC-CM active component–intersection target–hemostasis” network diagram to predict the primary constituents responsible for the hemostatic effect of GC-CM. The analysis identified the key core components as 1-monolinoleoyl-rac-glycerol, 2′,6′-dihydroxy 4′-methoxydihydrochalcone, 3-(4-formylaminobutyryl)pyridine, 6 h-thieno [3,2-f][1,2,4]triazolo [4,3-a][1,4]diazepine-6-acetic acid, 4-(4-chlorophenyl)-2,3,9-trimethyl-, 1,1-dimethylethyl ester, (6r)-, 1,3-dicyclohexylurea, and arachidonoyl ethanolamide. Molecular docking techniques confirmed that these six active components exhibit strong binding affinities with six core target proteins. Molecular dynamics simulations further demonstrated that the core targets, SRC and PIK3R1, maintained stability upon binding to the core components 1-mono-olinoleoyl-rac-glycerol and 2′,6′-dihydroxy 4′-methoxydihydrochalcone. Furthermore, prior research has demonstrated that arachidonoyl ethanolamide can enhance platelet activation and induce platelet aggregation [57,58,59]; however, the associations between other core components and hemostasis warrant further investigation.

While the present study offers valuable insights into the hemostatic mechanisms of GC-CM, certain limitations must be acknowledged. Firstly, although network pharmacology predictions have been validated through molecular docking and molecular dynamics simulations, further experimental validation is necessary. The computational approach is contingent upon the comprehensiveness of the database and the accuracy of prediction algorithms, which may introduce biases or fail to identify novel targets or pathways. Secondly, although initial in vitro experiments have demonstrated the hemostatic efficacy of GC-CM, additional in vivo experimental modeling is required to substantiate these findings. Future research should prioritize the experimental validation of core targets, such as SRC, AKT1, and PIK3R1, utilizing Western blotting in platelet activation models [60]. Concurrently, comprehensive in vivo hemostasis assessments, including tail bleeding time [61] and visceral bleeding model tests [62], should be conducted using animal bleeding models. We believe that GC-CM will be expected to be applied in the clinic as this research continues.

## 4. Materials and Methods

### 4.1. Source of Glenea cantor

Tree trunks infested by *Glenea cantor* were collected from Qingxiu Mountain Park in Nanning City (108°23′ E 22°47′ N) and placed in an insect rearing cage in the laboratory. When the adult *Glenea cantor* emerged from the wood, they were transferred into a glass bottle and provided with 3 fresh cottonwood branches (7 cm in length and 3 cm in diameter), and were reared indoors at a temperature of 25 ± 1 °C, a relative humidity of 70–80%, and a photoperiod of 14 L:10D to form an experimental population [63]. An appropriate number of female *Glenea cantor* that had emerged from the wood for 5 days from the 4th-generation experimental population were selected for backup.

### 4.2. Reagents and Instruments

Ultra-pure water was obtained from the Research Center for Differention and Development of TCM Basic Theory, Jiangxi University of Traditional Chinese Medicine (Nanchang, China). Acetonitrile (catalog number: 1499230-935, mass spectrometry grade, purity grade AR) and ammonium acetate (catalog number: 73594, mass spectrometry grade, purity grade AR) were purchased from Merck (Darmstadt, Germany). Methanol (catalog number: A456-4, mass spectrometry grade, purity grade AR) and ammonia water (catalog number: A470-500, mass spectrometry grade, purity grade AR) were purchased from Fisher Chemical (Waltham, MA, USA). An activated partial thromboplastin time (APTT) assay kit (tannic acid coagulation method, product number TC0306), a prothrombin time (PT) assay kit (one-stage method, product number TC0307), and sodium citrate anticoagulant (3.2% sterile, product number R10123) were purchased from Nanjing Juxionghua Biotechnology Co., Ltd. (Nanjing, China). Yunnan Baiyao (batch number ZDA2401) was purchased from Yunnan Baiyao Group Co., Ltd. (Kunming, China). A multifunctional microwave oven (Model M1-L213B) was purchased from Midea Group Co., Ltd. (Foshan, China). A numerical control ultrasonic (Model KQ-500DE) cleaner was purchased from Kunshan Ultrasonic Instrument Co., Ltd. (Kunshan, China). A UHPLC-Orbitrap Exploris™ 480 MS liquid chromatography–mass spectrometry system was purchased from Thermo Fisher Scientific (Waltham, MA, USA).

### 4.3. Experimental Animals

Male Sprague Dawley rats, aged 8 to 9 weeks and weighing between 250 and 280 g, were procured from Wuhan Myhalic Biotechnology Co., Ltd. (Wuhan, China). The production license for these experimental animals is SYXK (E) 2023-0104.

### 4.4. Preparation of GC-CM

GC-CM was prepared using the microwave-assisted thermal method. Five dried adult samples of *Glenea cantor* with comparable weights were obtained and then were thoroughly crushed in a mortar; 8 mL of ultrapure water was added to fully dissolve it, and then the obtained solution was added to a 100 mL beaker. Afterward, the beaker containing the mixture was placed in a microwave oven (700 W) and heated at high power for around 8 min to obtain a brown, crude GC-CM solid. Then, it was cooled down to room temperature. Lastly, at room temperature, the crude GC-CM was dissolved by adding 15 mL of ultrapure water, assisted by ultrasonic vibration, then filtered through paper to eliminate impurities, obtaining a uniform, light yellow solution.

### 4.5. In Vitro Coagulation Test

The procedures followed the guidelines outlined in the kit’s manual: orbital blood was collected from male Sprague Dawley rats (n = 6) into 0.109 mol/L sodium citrate anticoagulant (9:1 *v*/*v*). After gentle inversion, samples were centrifuged (3000 rpm, 10 min, 25 °C) to obtain platelet-poor plasma (PPP). Three experimental groups were established: (1) GC-CM group (0.1 mL *Glenea cantor* charcoal medicine, 0.0597 g/mL), (2) positive control (1 mg Yunnan Baiyao powder [64]), and (3) blank control (0.1 mL normal saline). For APTT measurement, 0.1 mL PPP was mixed with 0.1 mL APTT reagent (ellagic acid-based) and incubated at 37 °C for 5 min. Subsequently, 0.1 mL pre-warmed 0.025 mol/L CaCl_2_ was added to initiate clotting, with time recorded until fibrin formation via the manual tilt method. The activated partial thromboplastin time (APTT) assay is a diagnostic test that employs ellagic acid to activate factor XII at 37 °C. In this assay, partial thromboplastin substitutes for platelets, providing a catalytic surface essential for the coagulation process. The presence of calcium ions facilitates the conversion of fibrinogen into insoluble fibrin, allowing for the measurement of the time required for platelet-free plasma to coagulate. APTT is recognized as a sensitive and widely utilized screening test for assessing the intrinsic pathway of the coagulation cascade [65]. PT assessment followed a similar workflow: 0.07 mL PPP was combined with 0.14 mL PT reagent (tissue thromboplastin) and incubated at 37 °C (3 min), after which clotting time was measured identically. Determining prothrombin time (PT) involves the addition of an excess of tissue thromboplastin and calcium ions to the plasma sample under investigation, facilitating the conversion of prothrombin to thrombin. Subsequently, thrombin catalyzed the transformation of fibrinogen into fibrin. The duration required for the coagulation of platelet-deficient plasma was measured, providing insight into the prothrombin levels and the concentrations of factors V, VII, X, and fibrinogen within the plasma. This assay functions as a screening test for the extrinsic pathway of coagulation [66]. Both assays were performed in triplicate, and the results were expressed as mean ± SD. Activated partial thromboplastin time (APTT) and prothrombin time (PT) were independently assessed for the blank control group, the GC-CM group, and the positive control group. Subsequently, the intergroup differences were analyzed and compared. Prolonged APTT/PT indicates anticoagulant activity, while shortened values suggest procoagulant effects.

### 4.6. UHPLC-MS Analysis Conditions

#### 4.6.1. UHPLC Conditions

The samples were separated using a Vanquish LC ultra-high-performance liquid chromatography (UHPLC, Thermo Scientific, Shanghai, China) HILIC column (Waters, Shanghai, China). The column temperature was 25 °C, the flow rate was 0.5 mL/min, and the injection volume was 2 μL. Mobile phase A consisted of water + 25 mM ammonium acetate + 25 mM ammonia, and phase B was acetonitrile. The gradient elution program was as follows: 0–0.5 min, 95% B; 0.5–7 min, B linearly decreased from 95% to 65%; 7–8 min, B linearly decreased from 65% to 40%; 8–9 min, B maintained at 40%; 9–9.1 min, B linearly increased from 40% to 95%; 9.1–12 min, B maintained at 95%. During the entire analysis, the samples were kept in an automatic sampler at 4 °C. To avoid the effects of instrument signal fluctuations, samples were analyzed randomly. QC samples were inserted into the sample queue to monitor and assess the stability of the system and the reliability of the experimental data.

#### 4.6.2. Orbitrap Explorisrm™ 480 Mass Spectrometry Conditions

After separation using the Vanquish LC ultra-high-performance liquid chromatography (UHPLC) system, the samples were analyzed with an Orbitrap Explorisrm™ 480 mass spectrometer (Thermo Scientific, Shanghai, China), using electrospray ionization (ESI) in both positive and negative ion modes for detection. The settings for the ESI source and mass spectrometer were as follows: auxiliary gas 1 (Gas1) was set to 50, and auxiliary gas 2 (Gas2) to 2; the ion source temperature was set to 350 °C, with spray voltage (ISVF) of 3500 V in positive ion mode and 2800 V in negative ion mode; the mass range for the primary mass analysis was 70–1200 Da with a resolution of 60,000 and a scan time of 100 ms. The secondary scans used a segmented scanning method, covering a mass range of 70–1200 Da with a resolution of 60,000 and a scan time of 100 ms, with dynamic exclusion set to 4 s. The mass range for the primary mass analysis was 70–1200 Da with a resolution of 60,000 and a scan time of 100 ms, while the secondary scans used a segmented scanning method with a mass range of 70–1200 Da, a resolution of 60,000, and a scan time of 100 ms, featuring a dynamic exclusion time of 4 s.

#### 4.6.3. Data Analysis Process

The raw data were converted to .mzXML format using ProteoWizard (version 3.0.7414), and then peak alignment, retention time correction, and peak area extraction were performed using XCMS software(version 3.20). The data obtained from XCMS extraction were first subjected to metabolite identification and data preprocessing, including missing value filtering, missing value imputation (using KNN), and data filtering (deleting ion peaks with missing values > 50% and filtering features with RSD > 50%). Then, experimental data quality assessment and data analysis were performed.

### 4.7. Collection of Bioactive Components and Gene Targets of GC-CM

To ensure the comprehensiveness and accuracy of the data, chemical components exhibiting a match degree exceeding 95 in ultra-high performance liquid chromatography–mass spectrometry (UHPLC-MS) were screened for potential active constituents of GC-CM utilizing the Traditional Chinese Medicine Systems Pharmacology Database and Analysis Platform (TCMSP, https://www.tcmsp-e.com/tcmsp.php/, accessed on 24 September 2024). Following recommendations derived from the literature and the TCMSP database, the screening criteria were established with thresholds of oral bioavailability (OB) ≥ 30% and drug likeness (DL) ≥ 0.18 [67,68,69]. For the chemical constituents absent from the TCMSP database, their “Canonical SMILES” were obtained from PubChem (https://pubchem.ncbi.nlm.nih.gov/, accessed on 24 September 2024). Subsequently, these constituents were evaluated using Swiss ADME (http://www.swissadme.ch/, accessed on 24 September 2024) according to specific criteria: a “high” rating for gastrointestinal absorption (GI absorption) and a minimum of two “Yes” evaluations among the five drug likeness predictions (Lipinski, Ghose, Veber, Egan, Muegge) [70,71]. We entered the “Canonical SMILES” of the chosen active components into the SwissTargetPrediction tool (http://swisstargetprediction.ch/, accessed on 25 September 2024). From the resulting predictions, we selected targets with a probability greater than 0 to identify potential targets for GC-CM.

### 4.8. Collection of Potential Targets for Hemostasis

“Hemostasis” as the keyword and “Homo sapiens” as the species were used in the GeneCards database (https://www.genecards.org/, accessed on 26 September 2024), the Online Mendelian Inheritance in Man (OMIM) database (https://www.omim.org/, accessed on 26 September 2024), and the DrugBank database (https://go.drugbank.com/, accessed on 26 September 2024) to search for the disease utilization. In integrating disease targets, a higher relevance score signifies a stronger association between the target and the disease [72]; therefore, the targets with scores above the GeneCards median were selected. We transformed the target names retrieved from the Drugbank database using the Uniprot (https://www.uniprot.org/, accessed on 26 September 2024) database.

### 4.9. Construction of the “GC-CM Active Components–Intersection Targets–Hemostasis” Network

Venny 2.1 (https://bioinfogp.cnb.csic.es/tools/venny/, accessed on 28 September 2024) was used to determine the intersection between GC-CM active components and hemostasis potential targets, thereby identifying the common targets. A “GC-CM active components–intersection targets–disease” network was constructed using Cytoscape 3.9.1 to elucidate the potential molecular mechanisms underlying the interaction between GC-CM active components and the intersection targets. Subsequently, Cytoscape 3.9.1 was used to conduct a topological analysis of the hemostasis network processed by GC-CM to identify the core components associated with hemostasis among the potential active ingredients of GC-CM.

### 4.10. Construction of Protein–Protein Interaction (PPI) Network

To elucidate the interactions among various proteins within cells during the GC-CM-promoted hemostasis process and to comprehend the complexity and dynamics of the biological system, we utilized the STRING database (https://string-db.org/, accessed on 2 October 2024) to analyze the intersection targets of GC-CM and hemostasis. In this analysis, “Homo sapiens” was selected as the subject of study; the minimum interaction score with “highest confidence” (>0.900) was utilized for network target screening. The resulting protein–protein interaction (PPI) network data were visualized using CytoScape 3.9.1. Subsequently, Centiscape 2.2 in CytoScape3.9.1 was employed to determine the Degree Centrality (DC), Betweenness Centrality (BC), and Closeness Centrality (CC) values for each target. Hub genes were then identified based on the criterion that all three centrality parameters exceeded the median value, following two rounds of analysis.

### 4.11. Enrichment Analysis of GO and KEGG

The intersection targets of GC-CM and hemostasis were imported into the Metscape database (https://metascape.org/, accessed on 10 October 2024), specifying “Homo sapiens” as the organism. We conducted Gene Ontology (GO) and Kyoto Encyclopedia of Genes and Genomes (KEGG) enrichment analyses, applying a significance threshold of *p* < 0.01. The outcomes of these enrichment analyses elucidated the biological pathways and functions associated with the identified targets. Subsequently, bioinformatics tools (https://www.bioinformatics.com.cn/, accessed on 12 October 2024) were used to analyze and visualize the resultant data.

### 4.12. Molecular Docking

Based on the results of network pharmacology, the “GC-CM active components–intersection targets–hemostasis” network diagram was analyzed to identify the top six core active components with the highest degree values. Similarly, the protein interaction network diagram was examined to determine the top six core gene targets with the highest degree values after two rounds of screening. We obtained the “sdf” format of the six core active components using PubChem and converted it to “pdb” format using Open Babel GUI software(version 3.1.1). The protein structures in “pdb” format, corresponding to six core gene targets, were obtained from the RCSB Protein Data Bank (PDB) database (https://www.rcsb.org/, accessed on 23 October 2024). These structures pertain to the organism Homo sapiens and were determined using X-ray diffraction methods with resolutions of less than 3 Å. The specific PDB identifiers for the six proteins are as follows: SRC (PDB ID: 6F81), AKT1 (PDB ID: 4GV1), PIK3R1 (PDB ID: 7CIO), MAPK1 (PDB ID: 8AOJ), MAPK3 (PDB ID: 6GES), and PIK3CA (PDB ID: 8EXL). AutoDockTools (Version 1.5.7) was then used to remove water, add hydrogens to the proteins, add hydrogens to the active components, detect and select torsional bonds, and dock the processed proteins with the active components [73]. The binding energies for each docking result were then computed. Finally, Pymol 2.6 software was used to generate a 3D spatial environment image of the docking results.

### 4.13. Molecular Dynamics Simulation

Following the completion of molecular docking calculations for all compounds, molecular dynamics simulations were undertaken to further assess the stability of ligand–protein interactions. Based on the binding energy magnitude from the molecular docking results and the degree of each component, 100 ns molecular dynamics simulations were conducted on four sets of docking outcomes involving the core targets SRC and PIK3R1, as well as the core components 1-mono-linoleoyl-rac-glycerol and 2′,6′-dihydroxy-4′-methoxydihydrochalcone.

Molecular dynamics simulations were performed utilizing Gromacs 2020.4 [74] to assess the binding stability of the protein–ligand complex. The simulations employed the Amber99SB force field [75], and the system was solvated in a dodecahedral box containing TIP3P water molecules, subsequently neutralized with sodium and chloride ions [76]. Energy minimization was conducted until the force constant threshold was achieved. To simulate physiological conditions, the system was maintained at a constant temperature of 300 K and a pressure of 1 bar. Pre-equilibrium was achieved using a canonical ensemble (NVT) followed by a constant-pressure, constant-temperature (NPT) ensemble. The simulations were conducted incrementally over 100 ns with a time step of 1.25 fs, each step lasting 100 ps. The stability and convergence of the protein backbones within the docking complexes were evaluated by calculating the root mean square deviation (RMSD), root mean square fluctuation (RMSF), radius of gyration (RG), and solvent accessible surface area (SASA) using the built-in tools of Gromacs [77]. Analyzed data were visualized using GraphPad Prism Version 8.0.2 (263).

### 4.14. Statistical Analysis Methods

The experimental data were analyzed using IBM SPSS Statistics version 21.0, with results expressed as means ± standard deviation (SD). Normality of the data was confirmed through the Shapiro–Wilk and Kolmogorov–Smirnov tests [78,79], both yielding non-significant results (*p* > 0.05). Additionally, the chi-square test for variance homogeneity indicated no significant differences (*p* > 0.05). Subsequently, an independent-sample t-test was performed to compare the data between the two groups, with a significance threshold set at *p* < 0.05. Histogram analysis of the collected data was conducted using Origin 2021.

## 5. Conclusions

In conclusion, this study employed a comprehensive array of methodologies. In vitro coagulation experiments have yielded preliminary evidence indicating that GC-CM can reduce activated partial thromboplastin time (APTT) and prothrombin time (PT), thereby enhancing coagulation function and facilitating the overall hemostatic process. These findings are further supported by network pharmacology analyses. The in-depth analysis utilizing UHPLC-MS and network pharmacology revealed that the principal active constituents of GC-CM involved in the regulation of hemostasis are 1-monolinoleoyl-rac-glycerol and 2′,6′-dihydroxy 4′-methoxydihydrochalcone. The primary molecular targets identified include SRC, AKT1, and PIK3R1. Additionally, the key regulatory pathways implicated are platelet activation, complement and coagulation cascades, and the cGMP-PKG signaling pathway. Furthermore, molecular docking and molecular dynamics simulations demonstrate a significant affinity and binding stability between the primary active components of GC-CM and the key targets implicated in hemostasis. By demonstrating how a pest can be repurposed into a therapeutic resource, this work advances the sustainable utilization of ecological liabilities while providing a methodological blueprint for insect-derived drug discovery.

## Figures and Tables

**Table 1:** Results of the effect of GC-CM on APTT and PT (means ± SD, n = 3).

Groups	APTT (s)	PT (s)
blank control group	31.97 ± 1.90	12.77 ± 0.73
GC-CM group	24.93 ± 0.85 **	10.04 ± 0.70 **
positive control group	22.86 ± 1.42 **	9.68 ± 0.83 **

** *p* < 0.01 vs. blank control group. See List of Abbreviations for the meaning of APTT and PT.

**Table 2:** The top 20 active ingredients of GC-MC.

No.	Name	Degree	BC	CC
1	1-monolinoleoyl-rac-glycerol	23	0.15123	0.38356
2	2′,6′-dihydroxy 4′-methoxydihydrochalcone	20	0.13134	0.38043
3	3-(4-formylaminobutyryl)pyridine	13	0.08402	0.37333
4	6h-thieno [3,2-f][1,2,4]triazolo [4,3-a][1,4]diazepine-6-acetic acid, 4-(4-chlorophenyl)-2,3,9-trimethyl-, 1,1-dimethylethyl ester, (6r)-	9	0.05643	0.36939
5	1,3-dicyclohexylurea	7	0.04247	0.36745
6	Arachidonoyl ethanolamide	7	0.04247	0.36745
7	Galiellalactone	7	0.04247	0.36745
8	Neoabietic acid	7	0.04247	0.36745
9	1-(4-piperidinyl)-1,3-dihydro-2h-indol-2-one	6	0.03546	0.36649
10	4-(methylnitrosamino)-1-(3-pyridyl)-1-butanol	6	0.03546	0.36649
11	4-acetamidoantipyrin	6	0.03546	0.36649
12	alpha-ionone	5	0.02842	0.36554
13	(1r,5s)-8-methyl-8-azabicyclo [3.2.1]octan-3-amine	5	0.02842	0.36554
14	10-hydroxydecanoate	5	0.02842	0.36554
15	Dibutyl adipate	5	0.02842	0.36554
16	Dodecanoic acid, 12-[[(cyclohexylamino)carbonyl]amino]-	5	0.02842	0.36554
17	L-Fucose	5	0.02842	0.36554
18	N-acetyl-5-hydroxytryptamine	5	0.02842	0.36554
19	Primaquine	5	0.02842	0.36554
20	Pro-leu	5	0.02842	0.36554

See List of Abbreviations for the meaning of BC and CC.

**Table 3:** Topological parameters of 22 pivotal targets utilizing GC-CM hemostasis.

Target	Description	UniProt	DC	BC	CC
SRC	Proto-oncogene tyrosine-protein kinase Src	P12931	26	275.73	0.01176
AKT1	RAC-alpha serine/threonine-protein kinase	P31749	26	337.79	0.0119
PIK3R1	Phosphatidylinositol 3-kinase regulatory subunit alpha	P27986	22	87.27	0.01087
MAPK1	Mitogen-activated protein kinase 1	P28482	22	138.64	0.01099
MAPK3	Mitogen-activated protein kinase 3	P27361	21	154.09	0.01124
PIK3CA	Phosphatidylinositol 4,5-bisphosphate 3-kinase catalytic subunit alpha isoform	P42336	21	72.93	0.01075
TP53	Cellular tumor antigen p53	P04637	21	218.78	0.01087
PTK2	Focal adhesion kinase 1	Q05397	20	103.93	0.01042
STAT3	Signal transducer and activator of transcription 3	P40763	19	190.94	0.01087
PIK3CD	Phosphatidylinositol 4,5-bisphosphate 3-kinase catalytic subunit delta isoform	O00329	18	41.64	0.0101
PIK3CB	Phosphatidylinositol 4,5-bisphosphate 3-kinase catalytic subunit beta isoform	P42338	18	41.64	0.0101
JUN	Transcription factor Jun	P05412	18	204.33	0.01075
EGFR	Epidermal growth factor receptor	P00533	17	97.91	0.0101
RAF1	RAF proto-oncogene serine/threonine-protein kinase	P04049	15	43.79	0.00935
GRB2	Growth factor receptor-bound protein 2	P62993	14	83.64	0.00962
ESR1	Estrogen receptor	P03372	14	34.25	0.0101
FYN	Tyrosine-protein kinase Fyn	P06241	14	81.66	0.00909
LYN	Tyrosine-protein kinase Lyn	P07948	14	35.55	0.00935
TNF	Tumor necrosis factor	P01375	14	285.73	0.01031
CASP3	Caspase-3	P42574	12	90.93	0.0098
HIF1A	Hypoxia-inducible factor 1-alpha	Q16665	12	101.36	0.00962
MTOR	Serine/threonine-protein kinase mTOR	P42345	11	43.51	0.00909

See List of Abbreviations for the meaning of DC, BC and CC.

## Data Availability

The original contributions presented in the study are included in the article/Supplementary Materials; further inquiries can be directed to the corresponding authors.

## Supplementary Materials

The following supporting information can be downloaded at https://www.mdpi.com/article/10.3390/ph18040479/s1: Table S1: Two-hundred and two components with scores higher than 0.95.

## List of Abbreviations

GC-CM	*Glenea cantor* charcoal medicine
APTT	activated partial thromboplastin time
PT	prothrombin time
DC	Degree Centrality
BC	Betweenness Centrality
CC	Closeness Centrality
GO	Gene Ontology
KEGG	Kyoto Encyclopedia of Genes and Genomes
